# Identifying Similar Patterns of Structural Flexibility in Proteins by Disorder Prediction and Dynamic Programming

**DOI:** 10.3390/ijms160613829

**Published:** 2015-06-16

**Authors:** Aidan Petrovich, Adam Borne, Vladimir N. Uversky, Bin Xue

**Affiliations:** 1 Department of Physics, University of South Florida, Tampa, FL 33620, USA; E-Mail: aidan1@mail.usf.edu; 2 Department of Cell Biology, Microbiology, and Molecular Biology, School of Natural Sciences and Mathematics, College of Arts and Sciences, University of South Florida, Tampa, FL 33620, USA; E-Mail: bornea@mail.usf.edu; 3 Department of Molecular Medicine and USF Health Byrd Alzheimer’s Research Institute, Morsani College of Medicine, University of South Florida, Tampa, FL 33620, USA; E-Mail: vuversky@health.usf.edu; 4 Institute for Biological Instrumentation, Russian Academy of Sciences, Pushchino, Moscow Region 142290, Russian; 5 Department of Biology, Faculty of Science, King Abdulaziz University, P.O. Box 80203, Jeddah 21589, Saudi Arabia; 6 Laboratory of Structural Dynamics, Stability and Folding of Proteins, Institute of Cytology, Russian Academy of Sciences, St. Petersburg 194064, Russian

**Keywords:** intrinsic disorder, structural flexibility, disorder pattern, dynamic programming, dynamic time warping

## Abstract

Computational methods are prevailing in identifying protein intrinsic disorder. The results from predictors are often given as per-residue disorder scores. The scores describe the disorder propensity of amino acids of a protein and can be further represented as a disorder curve. Many proteins share similar patterns in their disorder curves. The similar patterns are often associated with similar functions and evolutionary origins. Therefore, finding and characterizing specific patterns of disorder curves provides a unique and attractive perspective of studying the function of intrinsically disordered proteins. In this study, we developed a new computational tool named IDalign using dynamic programming. This tool is able to identify similar patterns among disorder curves, as well as to present the distribution of intrinsic disorder in query proteins. The disorder-based information generated by IDalign is significantly different from the information retrieved from classical sequence alignments. This tool can also be used to infer functions of disordered regions and disordered proteins. The web server of IDalign is available at (http://labs.cas.usf.edu/bioinfo/service.html).

## 1. Introduction

Computational prediction is now a prevailing strategy in identifying intrinsically disordered proteins (IDPs) and intrinsically disordered regions (IDRs) for both individual proteins and entire proteomes [1,2,3]. By definition, IDPs/IDRs do not have rigid three-dimensional structures partially due to diminished hydrophobic interactions [4,5,6,7] determined by the specific amino acid compositions of IDPs and IDRs, which are typically depleted in hydrophobic, order-promoting residues, but are enriched in polar and charged disorder-promoting residues [8,9,10,11]. For this reason, information on the amino acid sequences and compositions of protein chains has been successfully used to predict if a given amino acid or a specific amino acid segment in a query protein is intrinsically disordered or not. Currently, the prediction accuracy of protein intrinsic disorder is reaching ~80% [1], which is becoming comparable with the accuracy of many low-resolution experimental techniques.

Although without rigid structures, IDPs/IDRs are highly abundant in nature and have critical biological functions. It was estimated that the fraction of IDPs/IDRs increases from ~20% in prokaryotic proteome to ~60% in eukaryotic proteomes [12,13,14]. Some prokaryotic proteomes also have higher fraction of IDPs/IDRs and were found to be associated with the extreme environmental conditions, at which the organisms thrive well [15,16]. The abundance of IDPs/IDRs in various proteomes is a strong indication that these proteins or regions perform important biological functions. Generally speaking, IDPs/IDRs play crucial roles in the processes of cell signaling and regulation [4,17,18]. In addition, IDPs/IDRs are also involved in many other functions. Post-translational modification sites are frequently within or near the IDRs [14,19,20,21,22,23,24,25,26,27,28]. Alternative splicing sites are also associated with IDRs [29,30,31,32]. Many long IDRs contain short hydrophobic-prone segments. These segments may undergo a disorder-to-order transition when forming complexes with specific binding partners [33]. The binding affinity with partners can also be tuned by IDRs [34,35]. IDPs/IDRs perform their functions through multiple mechanisms. In many cases, functionality is determined by specific amino acid sequences and compositions. In these cases, these functional regions can be recognized by sequence alignments. In other cases, the dynamics or the increased flexibility of disordered residues or regions is also critical. For example, disorder and related high structural flexibility facilitate the process of molecular recognition [36,37]. The dynamics of IDPs/IDRs is also a major contributor to the fuzziness of protein complexes [38].

Whether an amino acid residue or a protein segment is disordered or not can be evaluated by disorder scores generated by specific predictors. The predicted disorder scores of all residues in a query protein can be presented as a curve using the sequential index of amino acid as *x*-axis and disorder score as *y*-axis. The resulting curve is normally called disorder profile, per-residue disorder plot, or disorder curve. Disorder curve can be used to not only grade the structural flexibility of protein or its segments, but also infer functional roles of IDRs. For example, a “dip” within a segment that have high disorder scores indicates a short structure-prone motif in the middle of long disordered region, and this structure-prone motif may often act as a binding motif, such as molecular recognition feature (MoRF) [39,40] and ANCHOR-identified binding site (AIBS) [41]. Based on these observations, several computational tools have been developed to predict these disorder-based binding motifs [40,41,42,43,44].

The disorder curves of many proteins are similar to each other [45,46,47,48]. In addition, as evidenced by some of these studies, the similarity among disorder curves of query proteins often reflects common mechanisms of their function and evolution. The patterns of disordered curves are different from sequential patterns generated by traditional sequence alignment algorithms [49]. The disorder curves provide information on the structural flexibility, which may hardly be inferred from sequences directly. In a recent study, the peculiarities of disorder pattern were found to be critical for ion binding and the functions of heparinase II in *Pedobacter heparinus* [50]. In another recent study, comparison of disorder profiles was used to analyze regions that are involved in isoform-specific binding of tropomodulin (Tmod) to tropomyosin (TM), and to predict the residues that characterize isoform differences in binding [34]. In another related study, comparative analysis of disorder profiles of the wild type and the mutant forms of Tmod-1 and the wild type of Tmod-4 was used to define mutations that would affect the affinity of Tmod-1 to skeletal striated TM and make it similar to that of Tmod-4 [51]. A recent analysis on a dormancy-associated plant gene family DORMANCY 1/AUXIN Represses Protein (DRM1/ARP) revealed that these plant proteins can be grouped into six distinct classes based upon the similarity of their disorder profiles [52]. Similar analysis of another group of plant proteins, RPM1-interacting proteins 4 (RIN4) that belongs to the family of proteins containing nitrate-induced (NOI) domains and playing important roles in the plant immune responses to various pathogens, provided another proof that comparison of disorder curves facilitates functional annotation of proteins [53]. Additionally, disorder-based sequence alignments were used to show similarity of disorder distribution in several milk proteins, such as different casein classes [54], lactoperoxidases [55], and C- and N-lobes of lactoferrin [56]. Additionally, a new concept of *de novo* design of artificial IDPs was also recently brought into the scientific community [57]. Apparently, characterizing disorder patterns is a prerequisite for these new advancements in the IDP field.

However, to the best of our knowledge, none of the current computational tools in this field is specifically designed for comparative analysis of disorder patterns. Therefore, to fill this gap, we developed a novel computational tool to measure the similarity of different disorder curves by using dynamic programming. Dynamic programming has been broadly used in time series data analysis [58], sequence alignment [59], and string match [60]. In our study, dynamic programming was for the first time applied to compare disorder curves. It is expected that the results from this study provide new ideas to characterize patterns of intrinsic disorder and to infer functions associated with structural flexibility.

## 2. Results

### 2.1. Building up the Dataset

**Figure 1:**
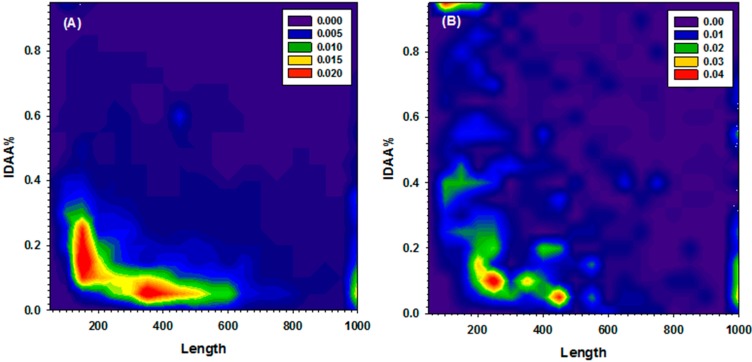
Abundance of proteins as a function of length and fraction of disordered residues (IDAA%) in both (**A**) Yeast and (**B**) DisProt datasets. All protein sequences longer than 1000 residues were merged with into the group of proteins of 1000 residues. The per-residue disorder score was calculated from PONDR-FIT. All residues of which the disorder score is higher than 0.5 were counted as disordered residues. The fraction of disordered residues is the ratio over the length of corresponding protein. Colors from purple, to blue, green, yellow, and red represents the increased abundance.

Figure 1 shows the distribution of proteins on a two-dimensional space formed by the length of protein sequences and the fraction of disordered residues in the sequence, for yeast proteome and for all disordered proteins in DisProt. Although the actual number of proteins is very different (6660 in yeast and 694 in DisProt), the overall distributions are similar. Most proteins have less than 500 residues, and less than 30% of disordered residues. It is also obvious that a small group of proteins in DisProt, which have less than 200 residues, is characterized by a high fraction of disordered residues (~100%). For the computational efficiency, 100 sequences shorter than 400 residues were randomly selected from each of these two sets, yeast proteome and DisProt proteins. These two dataset are referred to as Y100 and D100 dataset, respectively.

### 2.2. Gap Penalty

**Figure 2:**
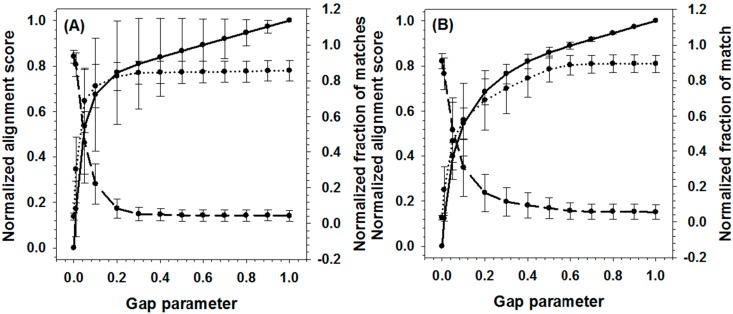
Influence of gap parameter on global matches for (**A**) Yeast dataset and (**B**) DisProt dataset. The solid line shows for normalized alignment score, while dash line and dotted lines represent normalized fraction of matches for two types of sequence pairs. Error bars present standard error. The first type of sequence pairs has lower similarity on their disorder curve and the calculated fraction of matches increases with gap penalty. The second type of sequence pairs is on the contrary. They have higher similarity on their disorder curves and their calculated fraction of matches decreases with the gap penalty.

For all sequence pairs in each of the datasets, their alignment scores increased when the gap penalty increased as shown in Figure 2. Nonetheless, the increment of alignment scores saturated after reaching the threshold values, indicating that the alignment is becoming stable. Therefore, the mean value of the normalized alignment scores over all sequence pairs in a dataset was used in Figure 2 to find the optimal threshold value of gap penalty for that dataset. The optimized gap penalty values for Y100 dataset and D100 dataset are slightly different, with the former being 0.3 and the latter being 0.5. In addition, mean value of normalized fraction of matches was also calculated to compare with the alignment score. It should be noted that all sequence pairs in each dataset can be divided into two sub-groups: (1) sequence pairs for which the fraction of matches decreases with the raising penalty score (typically, these are pairs with very low sequence similarities); and (2) sequence pairs for which the fraction of matches increases with the penalty score. Figure 2 demonstrates the presence of these two types of sequence pairs by showing two opposite trends in the correlation between fraction of matches and gap penalty scores. The lower standard errors within each group further validated the consistence of sequences within each group. This evidence added a second requirement on the selection of threshold value: The threshold value of gap penalty should have an intermediate value to balance the opposite influence on two sub-groups of sequence pairs. Therefore, after taking into consideration of all the factors, we chose 0.4 as the optimized gap penalty in all further studies.

### 2.3. Match Threshold

**Figure 3:**
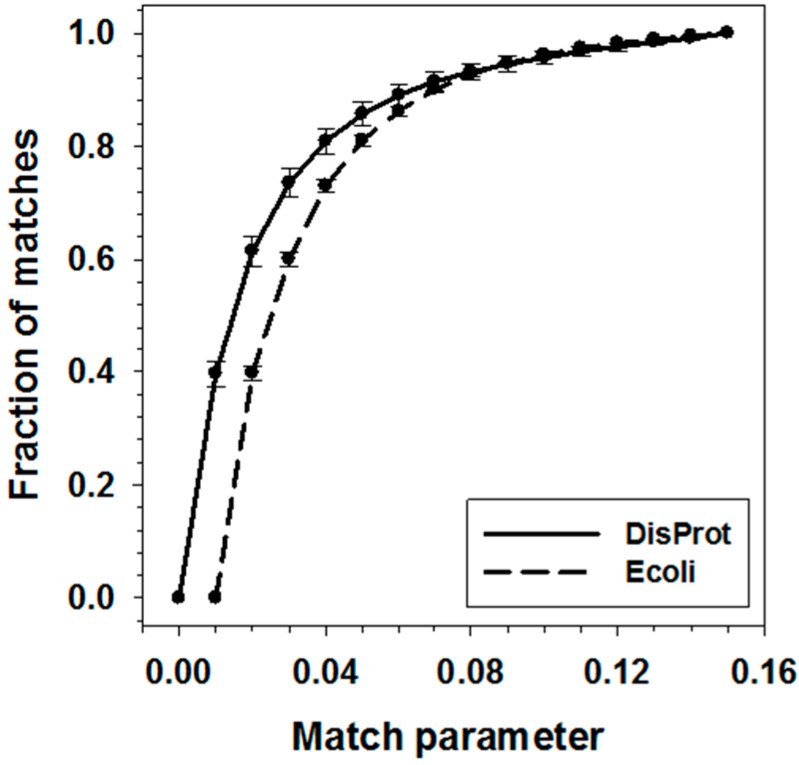
Match parameter influences fraction of identified matches. The averaged fraction of matches on one pair of sequences was calculated and then normalized in the datasets. Solid line and dash line are the correlation between match parameter and fraction of matches for DisProt and Yeast datasets, respectively.

After the alignment path being identified, the matches between data points were determined by comparing their pair-wise distance with the threshold of match score V_match_. The value of V_match_ will not affect the final alignment score, but only the fraction of identified matches. Conceivably, the larger the V_match_, the higher the fraction of matches. Figure 3 presented the analysis on the correlation between fraction of matches and V_match_ for both Y100 and D100 datasets. At the low end of each match threshold, the fractions of matches increased rapidly with the threshold value in both datasets. After reaching 0.05, the fractions became stabilized. Therefore, with the purpose of limiting the number of matched segments identified in the alignment, 0.05 was used as the threshold value for the matched data points in the application.

### 2.4. Examples/Applications

**Figure 4:**
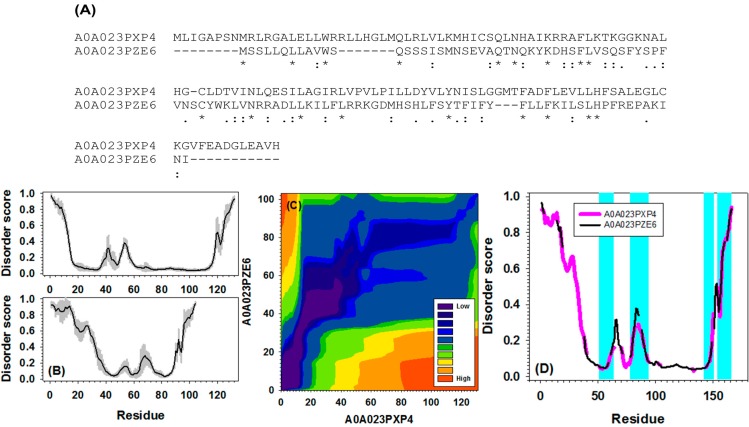
Identified alignment path for a sequence pair (Uniprot IDs: A0A023PXP4 and A0A023PZE6) from the Yeast dataset. (**A**) Traditional pair-wise sequence alignment. “*”, “:”, “.”, and “-” stand for identical amino acids, highly similar amino acids, similar amino acids, and gaps, respectively; (**B**) Original disorder predictions for A0A023PXP4 (**upper** panel) and A0A023PZE6 (**lower** panel). The gray shadow behind the disorder curves is estimated prediction error from PONDR-FIT; (**C**) Alignment path between two sequences identified by our newly developed package; (**D**) Alignment of disorder curves between A0A023PXP4 (pink) and A0A023PZE6 (black) along the alignment path. Many pairs of segments between pink and black curves overlap with each other. Only the pairs, of which the distance between two segments less than 0.05, were highlighted by cyan.

To examine the powerfulness and usefulness of this newly developed computational strategy, we performed several case studies. Figure 4 presents the similarity of disorder curves between two uncharacterized proteins from yeast (A0A023PXP4 and A0A023PZE6). These two proteins have different lengths, rather different sequences (see Figure 4A), and different fractions of disordered residues. However, they do have local similarity in their disorder profiles, such as the presence of the double-peak segments in the middle of their sequences, and the specific “dips” within their C-terminal tails (Figure 4B). The contour map in Figure 4C describes the similarity in more details. In the contour map, darker colors represent lower distance scores and therefore more similarity. Therefore, the region filled by darker colors connecting the first residue to the last residue tracks down a warping path and therefore represents the alignment path. It is clear that at the *N*-terminal ends, the alignment path is off-diagonal. In the region from 20 to 60 on *x*-axis and from 40 to 80 on *y*-axis, the alignment path becomes diagonal, representing matched curves. This region corresponds to the double-peak segments on both disorder curves. Afterwards, following another off-diagonal segment, the alignment path becomes narrow and diagonal in the range from ~110 to ~130 on *x*-axis and ~90 to ~100 on *y*-axis, indicating a highly matched curves at the C-terminal tails. By using the outputs from the identified alignment path, the original disorder curves were stretched and aligned in Figure 4D. The highlighted four short regions were characterized by similar patterns of the disorder distribution. By comparing the results of sequence alignment in Figure 4A, it is clear that these regions with matching disorder profiles have very limited sequence similarity.

**Figure 5:**
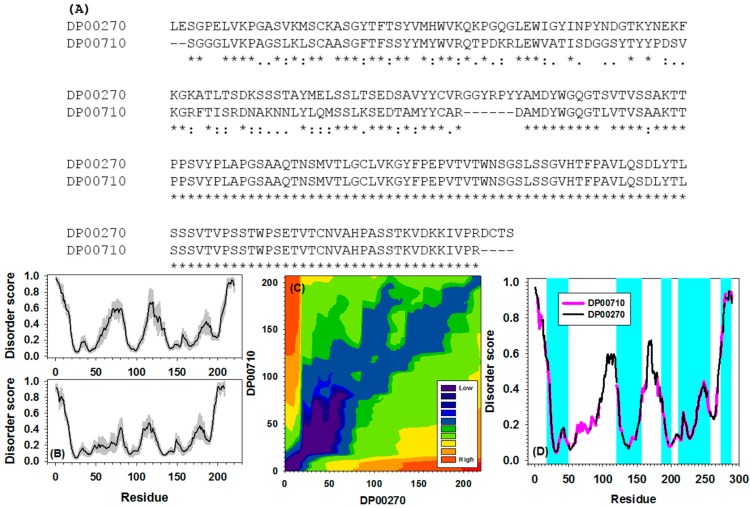
Identified alignment path for a sequence pair (Disprot IDs: DP00270 and DP00710) from the DisProt dataset. (**A**) Traditional pair-wise sequence alignment. “*”, “:”, “.”, and “-” stand for identical amino acids, highly similar amino acids, similar amino acids, and gaps, respectively; (**B**) Original disorder prediction for DP00270 (**upper** panel) and DP00710 (**lower** panel). The gray shadow behind the disorder curves is estimated prediction error; (**C**) Alignment path between two sequences identified by our newly developed package; (**D**) Alignment of disorder curves between DP00710 (pink) and DP00270 (black) along the alignment path. Only overlapped segment pairs of which the distance between two segments lower than 0.05 were highlighted by cyan.

Another example for the alignment of disorder curves between DP00270 (anti-ssDNA Fab DNA-1) and DP00710 (Fab fragment of immunoglobulin G1 MAK33 heavy chain) from the DisProt database is presented in Figure 5. These two proteins have very similar disorder curves in their C-terminal tails (Figure 5B). Actually, as shown in Figure 5A, the C-terminal tails of these two proteins have almost identical sequences. The alignment path in Figure 5C is almost completely diagonal but the color is lighter than that in Figure 4C. Multiple matched regions were identified in Figure 5D. Although the sequences become nearly identical starting at around residue 100, the disordered curves have less matches. The discrepancy comes from the predictive results of IUPred and will be explored further in the discussion section. An important conclusion from examples shown in both Figure 4 and Figure 5 is that the alignment of disorder curves provides additional information that may not be revealed by traditional sequence alignment.

At the next stage, we analyzed the usefulness of IDalign for the analysis of an important protein p53 and its homologues. Although this tumor suppressor is a well-known protein that does not require long introduction, some important information is provided below. The activity of this crucial transcription factor is modulated by various stress signals affecting genome integrity and cell proliferation. Activation of p53 triggers a complex cellular response regulating expression of genes involved in various biological processes, such as DNA repair, cell cycle progression, induction of apoptosis, response to cellular stress, senescence, *etc.* [61,62,63]. Some developmental abnormalities in animals are associated with the p53 deficiency [64]. Furthermore, the loss of p53 function is often related to the cancerous transformation of the cell [65]. In fact, cancers showing mutations in p53 are found in colon, lung, esophagus, breast, liver, brain, and in hemopoietic and reticuloendothelial tissues [65]. Human p53 is a 393 residue-long protein containing three functional regions, the N-terminal region, the central DNA Binding Domain (DBD), and the C-terminal region [62]. The N-terminal region can be further subdivided into TransActivation Domain 1 (TAD1) (residues 1–40), TAD2 (residues 40–60), and a Proline-Rich region, PR (residues 64–92). The C-terminal region contains a tetramerization or Oligomerization Domain (OD; residues 325–356), and a regulatory C-Terminal Domain (CTD; residues 356–393) [62,66]. Intrinsic disorder is known to be crucial for function of p53 [67,68,69], where, for example, the intrinsically disordered C-terminal region possesses a unique binding plasticity, being able not only to interact with various binding partners, but also to gain different structures in its bound form [70].

**Figure 6:**
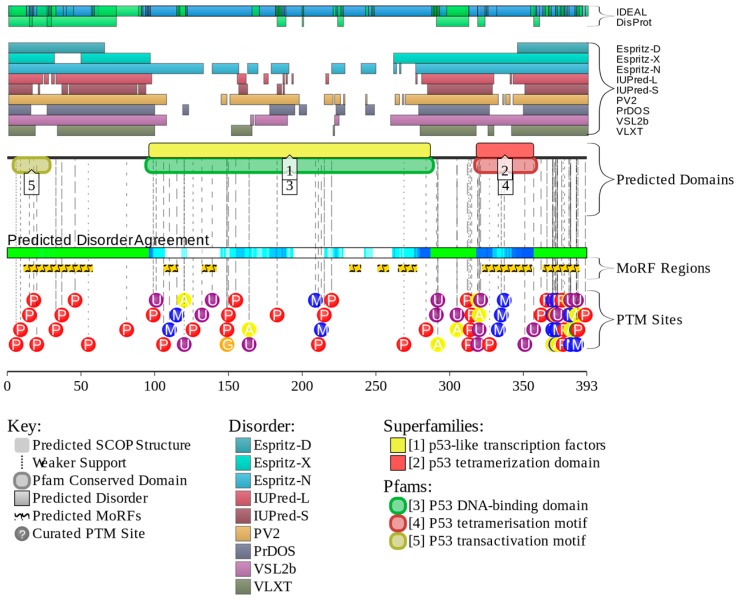
Evaluation of the functional intrinsic disorder propensity of human p53 (UniProt ID: P04637) by D^2^P^2^ database (http://d2p2.pro/) [71]. In this plot, top two lines represent annotated disordered regions in the DisProt and IDEAL databases. Next nine colored bars represent location of disordered regions predicted by different disorder predictors (Espritz-D, Espritz-N, Espritz-X, IUPred-L, IUPred-S, PV2, PrDOS, PONDR^®^ VSL2b, and PONDR^®^ VLXT, see keys for the corresponding color codes). Green-and-white bar in the middle of the plot shows the predicted disorder agreement between these nine predictors, with green parts corresponding to disordered regions by consensus. Yellow bar shows the location of the predicted disorder-based binding site (MoRF region), whereas colored circles at the bottom of the plot show location of sites of various posttranslational modifications (red—phosphorylation, blue—methylation, yellow—acetylation; orange—glycosylation; and violet—ubiquitylation).

For human p53, disorder evaluations together with important disorder-related functional information were retrieved from D^2^P^2^ database (http://d2p2.pro/) [71]. D^2^P^2^ is a database of predicted disorder that represents a community resource for pre-computed disorder predictions on a large library of proteins from completely sequenced genomes [71]. D^2^P^2^ database uses outputs of PONDR^®^ VLXT [8], IUPred [72], PONDR^®^ VSL2B [73,74], PrDOS [75], ESpritz [76], and PV2 [71]. This database is further enhanced by information on the curated sites of various posttranslational modifications and on the location of predicted disorder-based potential binding sites. Figure 6 represents the results of the application of this tool to human p53 and provides further support for the abundance and functional importance of intrinsic disorder in this protein. In fact, Figure 6 shows that this protein contains long disordered regions, which are enriched in potential disorder-based binding motifs and numerous sites of posttranslational modifications, PTMs. The fact that disordered domains/regions of human p53 are heavily enriched in various PTM sites is in agreement with the well-known notion that phosphorylation [77] and many other enzymatically catalyzed PTMs are preferentially located within the IDPRs [28].

**Figure 7:**
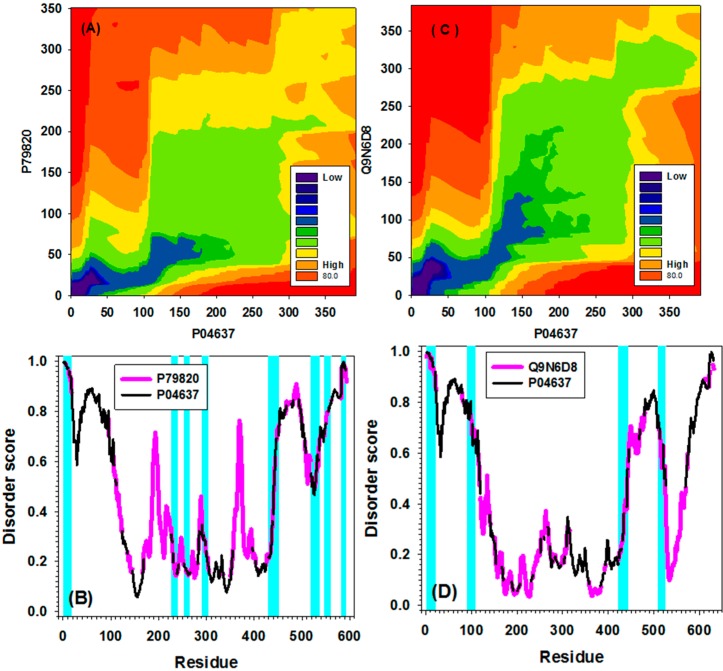
Identified alignment paths and alignments for sequence pairs between human p53 (Uniprot ID: P04637) and fish p53 (Uniprot ID: P79820) in (**A**,**B**), and between human p53 and fly p53 (Uniprot ID: Q9N6D8) in (**C**,**D**), respectively. (**A**,**C**) Alignment paths (contour maps) between two sequences in each of the sequence pairs were identified using our newly developed package; (**B**,**D**) Alignment of disorder curves along the alignment paths for two sequence pairs: P79820 (pink) and P04637 (black) in (**B**); Q9N6D8 (pink) and P04637 (black) in (**D**). Only overlapped segments of which the distance less than 0.05 were highlighted by cyan.

Figure 7 represents the results of the IDalign-based alignments of the disorder profiles of human p53 with its evolutionary distant homologues, p53 proteins from fish (UniProt ID: P79820) and fly (UniProt ID: Q9N6D8). In both cases, the resulting contour maps (Figure 7A,C), especially within the N-terminal regions of corresponding pairs, are asymmetric, with regions with darker colors that correspond to more similar sequence segments being located off-diagonal. Figure 7A,C also shows that there is another level of asymmetry, since the disorder-based similarity worsens while moving to the N- to the C-terminus, giving rise to the dark, off-diagonal, N-terminal regions and noticeably lighter, mostly on-diagonal, C-terminal regions. By using the outputs from the identified alignment paths, the original disorder curves were stretched and aligned (see Figure 7B,D). The highlighted short regions in Figure 7B,D correspond to sequence segments characterized by similar patterns of the disorder distribution. As expected, the number of these similar patterns is lower in more distant human-fly pair. The corresponding traditional sequence alignments are shown in Figure S1.

**Figure 8:**
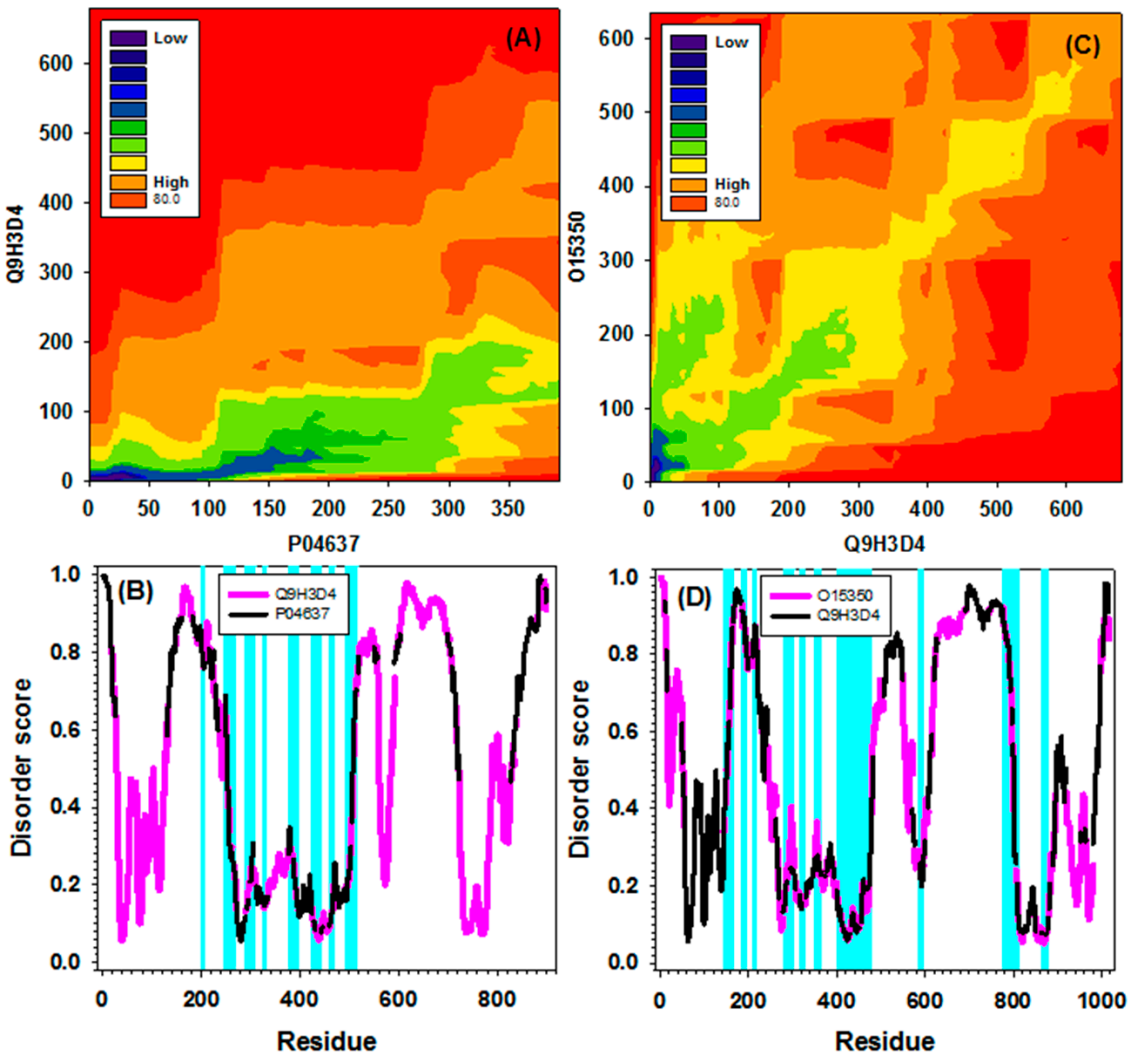
Identified alignment paths and alignments for sequence pairs between human p53 (Uniprot ID: P04637) and human p63 (Uniprot ID: Q9H3D4) in (**A**,**B**), and between human p63 and human p73 (Uniprot ID: O15350) in (**C**,**D**), respectively. (**A**,**C**) Alignment paths (contour maps) between two sequences in each of the sequence pairs were identified by our newly developed package; (**B**,**D**) Alignment of disorder curves along the alignment paths for two sequence pairs: Q9H3D4 (pink) and P04637 (black) in (**B**); O15350 (pink) and Q9H3D4 (black) in (**D**). Only overlapped segment pairs of which the distance less than 0.05 were highlighted by cyan.

The p53 protein is a member of an important protein family that includes p53, p63 (see [78,79]) and p73 [80]. Both p63 and p73 are structurally similar and functionally related to p53 [67]. The members of the p53 family are interlinked in a unique family-based signaling network that controls various aspects of cell life such as proliferation, differentiation, and death [63]. Both human p63 and p73 are almost two-fold longer than p53 and have 680 and 636 residues respectively. The domain organization of the members of p53-family is rather similar, and all three proteins have identifiable TAD, DBD and OD. In p63 and p73, there is an additional C-terminal sterile-α motif (SAM), which is required for p63 and p73 transcriptional activity, but they seem to lack the CTD found in p53 [63]. The various p53 family members have limited overall homology, but strong similarity in the DBD (approximately 60% between p53 and p63/p73 and approximately 85% between p63 and p73) [81,82]. Figure 8A,C represents the contour plots and the aligned profiles of human p53-p63 and p63-p73 pairs. Here, likely due to the large difference in the sequence lengths, the p53-p63 alignment is highly asymmetric, with the vast majority of darker regions being located off-diagonal (see Figure 8A). Although the contour plot representing the p63-p73 alignment is more symmetric (Figure 8C), this plot is characterized by noticeably lighter colors, which is expected due to the limited overall sequence homology of these two proteins. Figure 8B,D represents stretched and aligned disorder profiles of the human p53-p63 and p63-p73 pairs respectively. The highlighted short regions in Figure 8B,D correspond to sequence segments characterized by similar patterns of the disorder distribution. Note that these highlighted regions are concentrated mostly around the DBDs, reflecting higher levels of sequence/disorder pattern similarity in these domains in comparison with other regions. Again, the corresponding traditional pair-wise sequence alignments for human p53-p63 and p63-p73 pairs are shown in Figure S1.

### 2.5. Web Server

**Figure 9:**
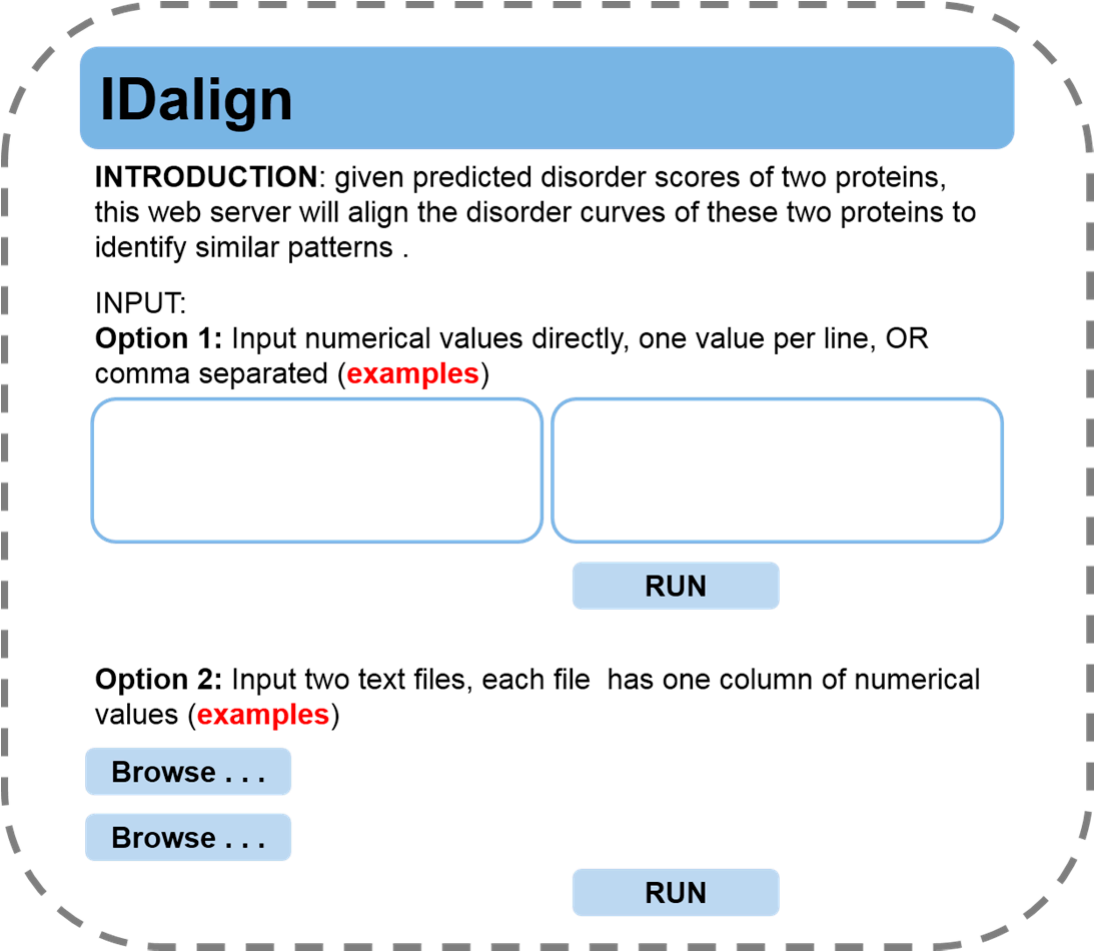
Layout the IDalign web server.

In order to facilitate the large-scale proteomic analysis of the common patterns of disorder curves, as well as the accurate positioning of the matched segments, we further developed a webserver, which can be accessed through the following link (http://labs.cas.usf.edu/bioinfo/service.html). The layout of this web server is shown in Figure 9. Users may choose one of the following two methods to input data. In the first method, the users may input comma-delimited disorder scores for two proteins. In the other method, the users may upload two data files that contain disorder scores in a single-column format. The output of the web server has three columns. The first column is the sequential index after alignment. The second and the third columns are disorder scores after alignment for the 1st and 2nd sets of input data, respectively. It should be noted that if two curves do not match to each other at a specific position, the score will be assigned as “−1”.

## 3. Discussions

It has been well recognized in the field of dynamic programming that both the gap penalty and the calculation of cost matrix may significantly shift the identified global matches. In various applications of dynamic time warping algorithms, the calculation of distance and cost matrix is extremely critical for the applicability of the algorithm. In this study, the calculation of distance is rather straight forward due to the nature of the problem. However, we did observe the significant influence of the gap penalty on the final output. When the gap penalty is low, a small increment may change the alignment score remarkably, as well as change the fraction of matches.

In addition, we observed the presence of two types of sequence pairs in both yeast and DisProt datasets. One type of the sequence pairs had low similarity of the corresponding disorder curves, and their overall fraction of matches increased with the gap penalty. The other type of sequence pairs had higher similarity of their disorder curves, and their fraction of matches decreased when the value of gap penalty was raised.

The aforementioned observations imposed rather strict criteria for the selection of gap penalties. Alignment score became saturated at higher values of gap penalty, indicating a stable alignment. In this meaning, the larger the gap penalty, the better the alignment. However, due to the presence of two types of sequence pairs, of which one type has decreased fraction of matches at higher penalty and the other has increased fraction of matches, a gap penalty of intermediate values may suit better to a general purpose alignment.

The disorder curves used in this study were generated by the PONDR-FIT, which is a meta-predictor built on six component predictors: PONDR-VLXT, PONDR-VSL2, PONDR-VL3, IUPred, FoldIndex, and TopIDP [83]. Most of the component predictors applied the sliding-window technique to take into consideration the influence of neighboring residues on the disorder score of the query residue, which is in the central of the sliding-window. Therefore, the disorder score of one residue is determined by all other residues inside the sliding-window that is normally 20 to 30 residues. In this respect, the IUPred [72] is very different from other component predictors. IUPred applies the pairwise amino acid interactions from next 100 amino acids along the sequence. Therefore, the calculated per-residue score from IUPred may be affected by the sequentially more distant residues. Consequently, the PONDR-FIT score may also be affected by the residues far away from the query residue. That is the reason why highly identical sequences in Figure 5D still have obviously different patterns of their disorder curves. To further validate our argument for the influence of IUpred on the alignment path, we tested the alignment between DP00710 and DP00270 using PONDR@VLXT scores [8] (Figure S2), which does not include the influence of long-range amino acids. It is clear that in the alignment of PONDR@VLXT scores, the influence of long-range amino acids is no longer present. Nonetheless, this difference shown in Figure 5D is advantageous since the result from IUPred takes into consideration the presence of long-range interactions, which are missed in other sliding-window methods. Therefore, the disorder curve from PONDR-FIT, which incorporates the results of IUPred, reflects partially the influence of long-range interactions on structural flexibility. That is also the reason why disorder-curve-based alignment provides additional information that is overlooked by sequence-alignment based methods.

## 4. Experimental Section

### 4.1. Datasets and Disorder Prediction

The entire proteome of *Saccharomyces cerevisiae* (strain ATCC 204508/S288c) was downloaded from UniProt (ftp://ftp.uniprot.org/pub/databases/uniprot/). This proteome contains 6740 protein sequences. After removing sequences shorter than 40 residues, the remaining dataset has 6660 proteins. All these protein sequences were predicted using PONDR-FIT [83] to get the per-residue disorder scores. In addition, the fraction of disordered residues was calculated for each sequence using 0.5 as threshold value for disordered residues. Finally, a subset of proteins of which the lengths are between 100 and 400, and the fractions of disordered residues are between 10% and 30% was randomly selected. The reasons for filtering out sequences shorter than 100 and longer than 400 amino acids are: (1) the prediction accuracies of terminal residues are normally lower than the accuracy of internal residues [84]. Since many predictors use sliding windows of 20~30 amino acids, the accuracies on the first and the last 20~30 residues in the sequence will be affected. Therefore, we chose 100 amino acids as the lower limit of the length of sequences in the dataset to ensure the overall acceptable prediction accuracy; (2) proteins are often organized in different domains. The length of a single domain is normally from tens of residues to around 400 amino acids. In other words, sequences longer than 400 amino acids may be composed of multiple domains and contain many linker regions. Therefore, we selected sequences shorter than 400 amino acids to build the dataset. The final dataset includes 100 sequences and is addressed as the Y100 dataset.

The second dataset used in this study is a subset of experimentally validated disordered proteins from DisProt [85]. DisProt has a total of 694 disordered protein sequences. All the sequences were also predicted by PONDR-FIT. Afterwards, 100 sequences with length in the range from 100 to 400 residues were selected to compose the D100 dataset.

### 4.2. Dynamic Programming

**Figure 10:**
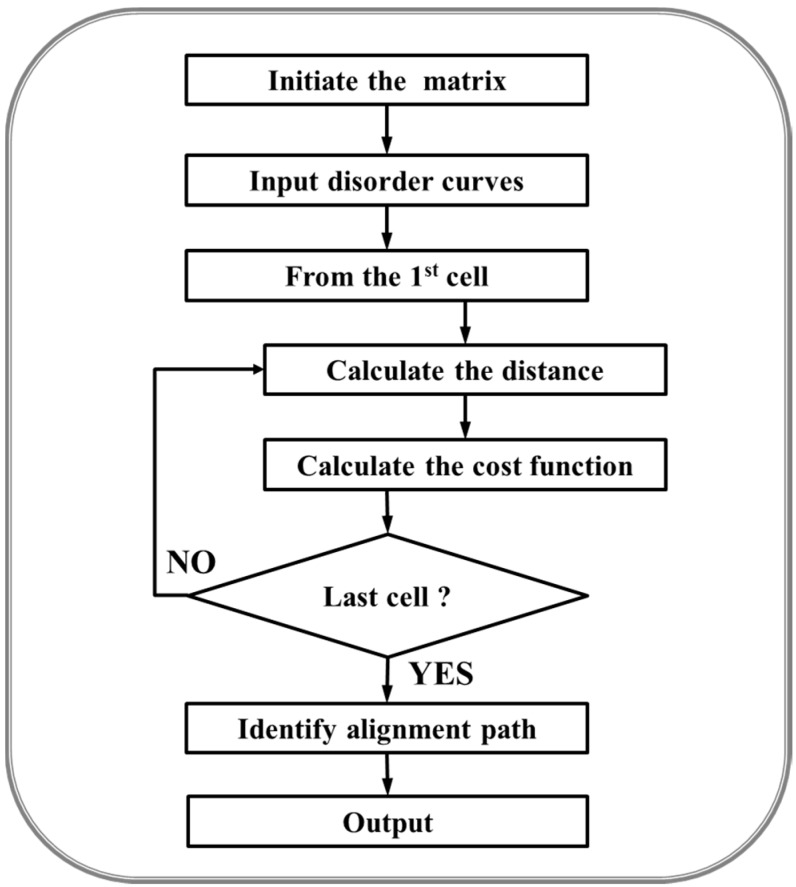
Pseudo code of the algorithm. Dynamic programming was applied to search for the similarity between two disorder curves. When initiating the matrix, the penalty score P was assigned to the first column and first row of the matrix. Then the data points on the disorder curves were uploaded into the 2nd column and 2nd row of the matrix. Next, the distance and cost function were calculated using the formula described in the method section starting from the first vacant cell. After completing the calculation for all cells in the matrix, the alignment path was identified starting from the last cell to the first cell by connecting cells with lower cost function values.

Assume the first disorder curve has N data points (x_1_, x_2_, . . ., x_N_), and the second one has M data points (y_1_, y_2_, . . ., y_M_). The purpose of this study is to calculate the pair-wise distance between the data points on two different curves and finally to evaluate the similarity between two curves. We designed an algorithm similar to dynamic time warping (DTW) [86] to compare the disorder curves. The Euclidian distance between any two data points from two different curves was calculated as f_i,,j_ = |x_i_ − y_j_|, I = (1, N) and j = (1, M). The cost function in the algorithm is F_i,j_ = min (f_i-1, j-1_, f_i-1, j_, f_i, j-1_) + f_i, j_. In addition, we also introduced a gap penalty score P when initializing the cost function. The gap penalty serves as a global constraint and does influence the results of identified global matches. The pseudo code for the implementation of the algorithm is shown in Figure 10.

### 4.3. Fraction of Matches

The data points in this study are per-residue disorder scores, which ranges from 0 to 1. The result from above-mentioned dynamic programming analysis is an alignment path (or warping path as used in DTW). Therefore, the distances between data points from two curves along this path can be calculated. The distances can also be compared to a threshold value V_match_. If the distance between two data points is less than V_match_, these two data points are considered to be a pair of matches. Furthermore, the fraction of matched data points for a pair of curves can be calculated as f_M_ = 2 × N_match_/(N_1_ + N_2_). N_match_ is the number of matched data points. N_1_ and N_2_ are the lengths of two curves.

## Supplementary Materials

Supplementary materials can be found at http://www.mdpi.com/1422-0067/16/06/13829/s1.
