# Supplementary material for: Identifying Similar Patterns of Structural Flexibility in Proteins by Disorder Prediction and Dynamic Programming
Source: Int J Mol Sci. 2015 Jun 16;16(6):13829–49. doi: 10.3390/ijms160613829 (PMC4490526; doi:10.3390/ijms160613829)
Supplement: Supplementary file 1 [file ijms-16-13829-s001.pdf]

Supplementary Information

```
P04637          -------------MEEPQSDPSVEPP-----------------------LSQETFSDLWK 24
Q9H3D4          MNFETSRCATLQYCPDPYIQRFVETPAHFSWKESYYRSTMSQSTQTNEFLSPEVFQHIWD 60
                               :*  :  **.*                       ** *.*..:*.

P04637          LLPE----------NNVLSPLPSQAMDDLMLSPDDIEQWFTEDPGP-------------- 60
Q9H3D4          FLEQPICSVQPIDLNFVDEPSEDGATNKIEISMDCIRMQDSDLSDPMWPQYTNLGLLNSM 120
                :* :          * * .*  . * :.: :* * *.   :: ..*

P04637          ------DEAPRMPEAAPPVAPAPAAPTPAAPAPAPSWPLSSS--VPSQKTYQGSYGFRLG 112
Q9H3D4          DQQIQNGSSSTSPYNTDHAQNSVTAPSPYAQPSSTFDALSPSPAIPSNTDYPGPHSFDVS 180
                      ..:.  *  :  .  : :**:* * ..:.  .**.*  :**:. * *.:.* :.

P04637          FLHSGTAKSVTCTYSPALNKMFCQLAKTCPVQLWVDSTPPPGTRVRAMAIYKQSQHMTEV 172
Q9H3D4          FQQSSTAKSATWTYSTELKKLYCQIAKTCPIQIKVMTPPPQGAVIRAMPVYKKAEHVTEV 240
                * :*.****.* ***. *:*::**:*****:*: * :.** *: :***.:**:::*:***

P04637          VRRCPHHERCSDSD--GLAPPQHLIRVEGNLRVEYLDDRNTFRHSVVVPYEPPEVGSDCT 230
Q9H3D4          VKRCPNHELSREFNEGQIAPPSHLIRVEGNSHAQYVEDPITGRQSVLVPYEPPQVGTEFT 300
                *:***:** . : :   :***.******** :.:*::*  * *:**:******:**:: *

P04637          TIHYNYMCNSSCMGGMNRRPILTIITLEDSSGNLLGRNSFEVRVCACPGRDRRTEEENLR 290
Q9H3D4          TVLYNFMCNSSCVGGMNRRPILIIVTLETRDGQVLGRRCFEARICACPGRDRKADEDSIR 360
                *: **:******:********* *:***  .*::***..**.*:********:::*:.:*

P04637          KKGEPHHELPPGSTKRALPNNTS---SSPQPKKKPLDGEYFTLQIRGRERFEMFRELNEA 347
Q9H3D4          KQQVSDSTKNGDGTKRPFRQNTHGIQMTSIKKRRSPDDELLYLPVRGRETYEMLLKIKES 420
                *:  ..     ..***.: :**     :.  *::. *.* : * :**** :**: :::*:

P04637          LELKD-----------------------AQAGKEPGGSRAHSS----------HLKSKKG 374
Q9H3D4          LELMQYLPQHTIETYRQQQQQQHQHLLQKQTSIQSPSSYGNSSPPLNKMNSMNKLPSVSQ 480
                *** :                        *:. :. .* .:**          :* * .

P04637          QSTSRHKKLMFKTEGPDSD----------------------------------------- 393
Q9H3D4          LINPQQRNALTPTTIPDGMGANIPMMGTHMPMAGDMNGLSPTQALPPPLSMPSTSHCTPP 540
                  ..:::: :  *  **.

P04637          ------------------------------------------------------------
Q9H3D4          PPYPTDCSIVSFLARLGCSSCLDYFTTQGLTTIYQIEHYSMDDLASLKIPEQFRHAIWKG 600

P04637          ------------------------------------------------------------
Q9H3D4          ILDHRQLHEFSSPSHLLRTPSSASTVSVGSSETRGERVIDAVRFTLRQTISFPPRDEWND 660

P04637          --------------------
Q9H3D4          FNFDMDARRNKQQRIKEEGE 680
```

(A)

Figure S1. *Cont.*

```
Q9H3D4              MNFETSRCATLQYCPDPYIQRFVETPAHFSWKESYYRSTMSQSTQTNEFLSPEVFQHIWD 60
O15350              ---------------------------------------MAQSTATSPDGG-TTFEHLWS 20
                                                           *:*** *.   .  .*:*:*.

Q9H3D4              FLEQPICSVQPIDLNFVDEPSEDGATNKIEISMDCIRMQDSDLSDPMWPQYTNLGLLNSM 120
O15350              SLEP---DSTYFDLPQSSRGNNE-VVGGTDSSMDVFHLEG--MTTSVMAQFN---LLSST 71
                     **    .   :**   .. .:: ...  : *** ::::.  :: .: .*:.   **.*

Q9H3D4              DQQIQNGSSSTSPYNTDHAQNSVTAPSPYAQPSSTFDALSPSPAIPSNTDYPGPHSFDVS 180
O15350              MDQMSSRAASASPYTPEHAA-SVPTHSPYAQPSSTFDTMSPAPVIPSNTDYPGPHHFEVT 130
                     :*:.. ::*:***..:**  **.: ***********::**:*.*********** *:*:

Q9H3D4              FQQSSTAKSATWTYSTELKKLYCQIAKTCPIQIKVMTPPPQGAVIRAMPVYKKAEHVTEV 240
O15350              FQQSSTAKSATWTYSPLLKKLYCQIAKTCPIQIKVSTPPPPGTAIRAMPVYKKAEHVTDV 190
                    ***************. ****************** **** *:.**************:*

Q9H3D4              VKRCPNHELSREFNEGQIAPPSHLIRVEGNSHAQYVEDPITGRQSVLVPYEPPQVGTEFT 300
O15350              VKRCPNHELGRDFNEGQSAPASHLIRVEGNNLSQYVDDPVTGRQSVVVPYEPPQVGTEFT 250
                    *********.*:***** **.*********. :***:**:******:*************

Q9H3D4              TVLYNFMCNSSCVGGMNRRPILIIVTLETRDGQVLGRRCFEARICACPGRDRKADEDSIR 360
O15350              TILYNFMCNSSCVGGMNRRPILIIITLEMRDGQVLGRRSFEGRICACPGRDRKADEDHYR 310
                    *:**********************:*** *********.**.***************  *

Q9H3D4              KQQVS--DSTKNGDGTKRPFRQNTHGIQMT--SIKKRRSPDDELLYLPVRGRETYEMLLK 416
O15350              EQQALNESSAKNGAASKRAFKQSPPAVPALGAGVKKRRHGDEDTYYLQVRGRENFEILMK 370
                    :**.   .*:*** .:**.*:*.. .:     .:****  *::  ** *****.:*:*:*

Q9H3D4              IKESLELMQYLPQHTIETYRQQQQQQHQHLLQKQTSIQSPSSYGNSSPPLNKMN-SMNKL 475
O15350              LKESLELMELVPQPLVDSYRQQQQ-----LLQRPSHLQPPS-YGPVLSPMNKVHGGMNKL 424
                    :*******: :**  :::******     ***: : :*.** **   .*:**:: .****

Q9H3D4              PSVSQLIN--PQQRNALTPTTIPDGMGANIPMMGTHMPMAGDMNGLSPTQALPPPLSMPS 533
O15350              PSVNQLVGQPPPHSSAATPNLGPVGPGM-LNNHGHAVPANGEMS------SSHSAQSMVS 477
                    ***.**:.  * : .* **.  * * *  :   *  :*  *:*.      :  ..  ** *

Q9H3D4              TSHCTPPPPYPTDCSIVSFLARLGCSSCLDYFTTQGLTTIYQIEHYSMDDLASLKIPEQF 593
O15350              GSHCTPPPPYHADPSLVSFLTGLGCPNCIEYFTSQGLQSIYHLQNLTIEDLGALKIPEQY 537
                     ********* :* *:****: ***..*::***:*** :**:::: :::**.:******:

Q9H3D4              RHAIWKGILDHRQLHEFSSPSHLLRTPSSASTVSVGSS-ETRGERVIDAVRFTLRQTISF 652
O15350              RMTIWRGLQDLKQGHDYSTAQQLLRS-SNAATISIGGSGELQRQRVMEAVHFRVRHTITI 596
                    * :**:*: * :* *::*:..:***: *.*:*:*:*.* * : :**::**:* :*:**::

Q9H3D4              PPR-------DEWNDFNFDMDARRNKQQRIKEEGE----- 680
O15350              PNRGGPGGGPDEWADFGFDLPDCKARKQPIKEEFTEAEIH 636
                    * *       *** **.**:   : ::* ****
```

(**B**)

Figure S1. *Cont.*

```
P04637          MEEPQSDPSVEPPLSQETFSDLWKLLPENNVLSPLPSQAMDDLMLSPDDIEQWFTEDPGP 60
P79820          -----MDPVPDLPESQGSFQELWETVS-YPPLETLSLPTVNEPTGSWVATGDMFLLDQDL 54
                      **  : * ** :*.:**: :.    *..*.  ::::   *     : *  * .

P04637          DEAPRMPEAAPPVAPAPAAPTPAAPAPAPSWPLSSSVPSQKTYQGSYGFRLGFLHSGTAK 120
P79820          SGTFDDKIFDIPIEPVPTN----EVNPPP-----TTVPVTTDYPGSYELELRFQKSGTAK 105
                . :        *: *.*:        *.*     ::**  . * *** :.* * :*****

P04637          SVTCTYSPALNKMFCQLAKTCPVQLWVDSTPPPGTRVRAMAIYKQSQHMTEVVRRCPHHE 180
P79820          SVTSTYSETLNKLYCQLAKTSPIEVRVSKEPPKGAILRATAVYKKTEHVADVVRRCPHHQ 165
                ***.*** :***::******.*::: *.. ** *: :** *:**:::*:::********:

P04637          RCSDSDGLAPPQHLIRVEGNLRVEYLDDRNTFRHSVVVPYEPPEVGSDCTTIHYNYMCNS 240
P79820          ---NEDSVEHRSHLIRVEGSQLAQYFEDPYTKRQSVTVPYEPPQPGSEMTTILLSYMCNS 222
                   :.*.:   .*******.  .:*::*  * *:**.******: **: ***   .*****

P04637          SCMGGMNRRPILTIITLEDSSGNLLGRNSFEVRVCACPGRDRRTEEENLRKKGEPHHELP 300
P79820          SCMGGMNRRPILTILTLET-EGLVLGRRCFEVRICACPGRDRKTEEESRQKTQPKKRKVT 281
                **************:***  .* :***..****:********:****. :*.   ::::.

P04637          PGSTKRALPNNTSSSPQPKKKPLDGEYFTLQIRGRERFEMFRELNEALELKDAQAGKEPG 360
P79820          PNTSSSKRKKSHSSGEEEDNR----EVFHFEVYGRERYEFLKKINDGLELLEKESK---- 333
                *.::.     :. **. : .::     * * ::: ****:*:::::*:.*** : ::

P04637          GSRAHSSHLKSKKGQSTSRHKKLMFKTEGPDSD 393
P79820          -SKNKDS------GMVPSSGKKLKSN------- 352
                 *: :.*      *  .*  ***  :
```

(**C**)

```
P04637          MEEPQSDPSVEPPLSQETFSDLWKLLPENNVLSPLPSQAMDDLMLSPDDIEQWFTEDPGP 60
Q9N6D8          -------MYISQPMS-------WHKESTDSEDDSTEVDIKEDIPKTVEVSGSELTTEP-- 44
                         :. *:*       *:  . :.  ..   :  :*:  : :   . :* :*

P04637          DEAPRMPEAAPPVAPAPAAPTPAAPAPAPSWPLSSSVPSQKTYQGSYGFRLGFLHSGTAK 120
Q9N6D8          --MAFLQGLNSGNLMQFSQQSVLREMMLQDIQIQANTLPKLENHNIGGYCFSMVLDEPPK 102
                   . :    .       :  :         .  :.:.. .:   :.  *: :.:: . ..*

P04637          SVTCTYSPALNKMFCQLAKTCPVQLWVDSTPPPGTRVRAMAIYKQSQHMTEVVRRCPHHE 180
Q9N6D8          SL-WMYSIPLNKLYIRMNKAFNVDVQFKSKMPIQP-LNLRVFLCFSNDVSAPVVRCQNHL 160
                *:   ** .***:: :: *:  *:: ..*. *   . :.  .:   *:.::  * ** :*

P04637          RCS--DSDGLAPPQHLIRVEGNLRVEYLDDRN---TFRHSVVVPYEPP----EVGSDCTT 231
Q9N6D8          SVEPLTANNAKMRESLLRSENPNSVYCGNAQGKGISERFSVVVPLNMSRSVTRSGLTRQT 220
                  .   ::.    : *:* *.   *    : :.   : *.***** : .     . *    *

P04637          IHYNYMCNSSCMGGMNRRPILTIITLEDSSGNLLGRNSFEVRVCACPGRDRRTEEENLRK 291
Q9N6D8          LAFKFVCQNSCIG---RKETSLVFCLEKACGDIVGQHVIHVKICTCPKRDRIQDERQLNS 277
                : ::::*:.**:*   *:    :: **.:.*::*:: :.*::*:** ***  :*.:*..

P04637          KG-----EPHHELPPGSTKRALPNNTSSSPQPKKKPLDGEYFTLQIRGRERFEMFRELNE 346
Q9N6D8          KKRKSVPEAAEEDEPSKVRRCIAIKTEDTESNDSRDCDDSAAEWNVS-RTPDGDYRLAIT 336
                *      *. .*  *...:*.:. :*..: . ..:  *..    ::  *     :*

P04637          ALELKDAQAGKEPGGSRAHSSHLKSKKGQSTSRHKKLMFKTEGPDSD-- 393
Q9N6D8          CPNKEWLLQSIEGMIKEAAAEVLRNPNQENLRRHANKLLSLKKRAYELP 385
                . : :    . *   ..* :. *:. : :.  ** : ::. :    :
```

(**D**)

Figure S1. Pair-wise sequence alignment. "*", ":", ".", and "-" stand for identical amino acids, highly similar amino acids, similar amino acids, and gaps, respectively. (**A**) Human P53 (Uniprot ID: P04637) *vs.* human P63 (Uniprot ID: Q9H3D4); (**B**) Human P63 (Uniprot ID: Q9H3D4) *vs.* human P73 (Uniprot ID: O15350); (**C**) Human P53 (Uniprot ID: P04637) *vs.* fish P53 (Uniprot ID: P79820); (**D**) Human P53 (Uniprot ID: P04637) *vs.* Fly P53 (Uniprot ID: Q9N6D8).

(A)

```
DP00270      LESGPELVKPGASVKMSCKASGYTFTSYVMHWVKQKPGQGLEWIGYINPYNDGTKYNEKF
DP00710      --SGGGLVKPAGSLKLSCAASGFTFSSYYMYWVRQTPDKRLEWVATISDGGSYTYYPDSV
               **  ****..*:*:** ***:**:** *:**:*.*.: ***:. *.  .. * * :..

DP00270      KGKATLTSDKSSSTAYMELSSLTSEDSAVYYCVRGGYRPYYAMDYWGQGTSVTVSSAKTT
DP00710      KGRFTISRDNAKNNLYLQMSSLKSEDTAMYYCAR------DAMDYWGQGTLVTVSAAKTT
             **: *:: *::... *:::***.***:*:***.*       ********* ****:****

DP00270      PPSVYPLAPGSAAQTNSMVTLGCLVKGYFPEPVTVTWNSGSLSSGVHTFPAVLQSDLYTL
DP00710      PPSVYPLAPGSAAQTNSMVTLGCLVKGYFPEPVTVTWNSGSLSSGVHTFPAVLQSDLYTL
             ************************************************************

DP00270      SSSVTVPSSTWPSETVTCNVAHPASSTKVDKKIVPRDCTS
DP00710      SSSVTVPSSTWPSETVTCNVAHPASSTKVDKKIVPR----
             ************************************
```

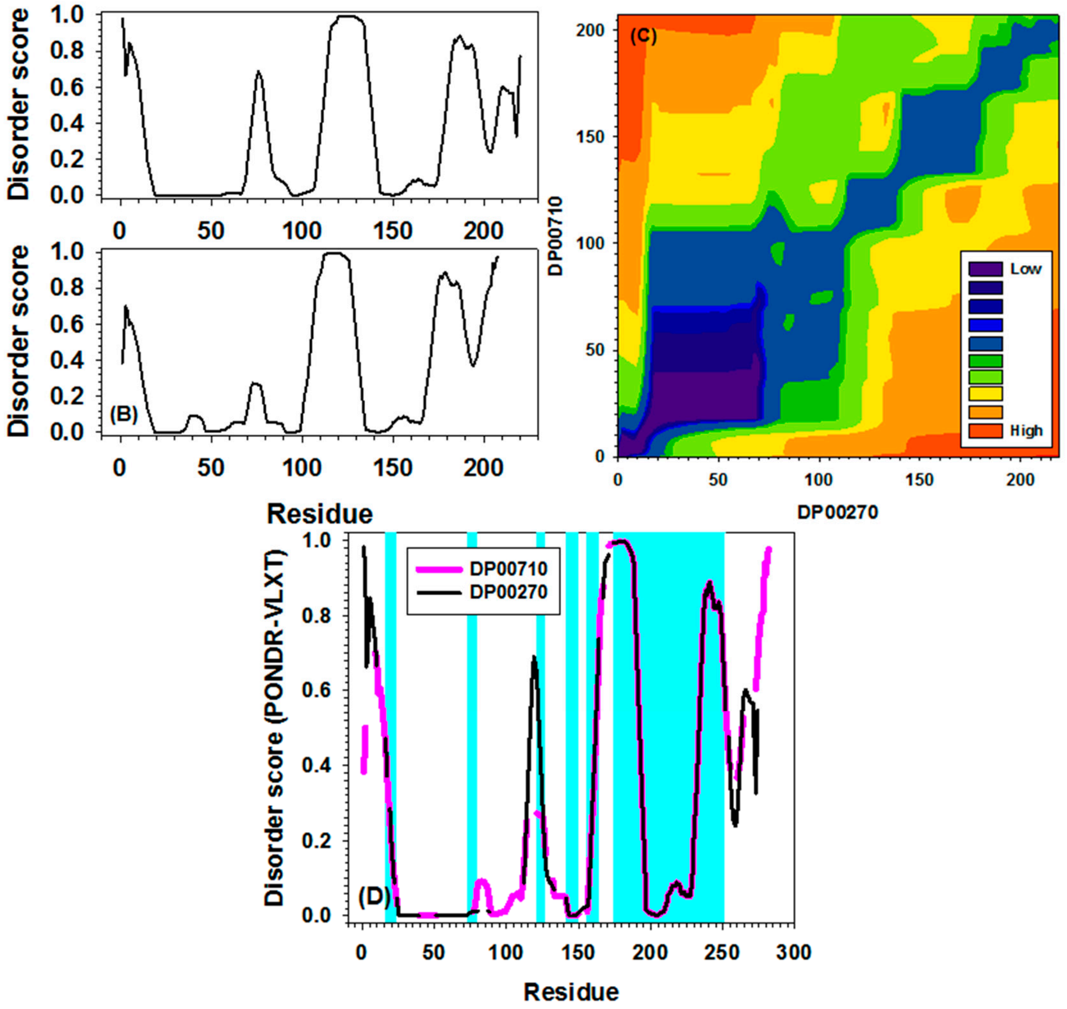

Figure S2. Alignment analysis for DP00270 and DP00710. (**A**) Pair-wise sequence alignment; "*", ":", ".", and "-" stand for identical amino acids, highly similar amino acids, similar amino acids, and gaps, respectively; (**B**) Disorder prediction for DP00270 (**upper** panel) and DP00710 (**lower** panel) by PONDR® VLXT; (**C**) Identified alignment path between DP00270 and DP00710; and (**D**) Alignment of disorder curves between DP00710 (pink) and DP00270 (black) along the alignment path. Only overlapped segment pairs of which the distance between two segments lower than 0.05 were highlighted by cyan.
